# Next steps for solvent-based CO_2_ capture; integration of capture, conversion, and mineralisation

**DOI:** 10.1039/d2sc00220e

**Published:** 2022-05-19

**Authors:** David J. Heldebrant, Jotheeswari Kothandaraman, Niall Mac Dowell, Lynn Brickett

**Affiliations:** Pacific Northwest National Laboratory Richland WA USA david.heldebrant@pnnl.gov; Washington State University Pullman WA USA; Imperial College London London England; US Department of Energy, Office of Fossil Energy USA

## Abstract

In this perspective, we detail how solvent-based carbon capture integrated with conversion can be an important element in a net-zero emission economy. Carbon capture and utilization (CCU) is a promising approach for at-scale production of green CO_2_-derived fuels, chemicals and materials. The challenge is that CO_2_-derived materials and products have yet to reach market competitiveness because costs are significantly higher than those from conventional means. We present here the key to making CO_2_-derived products more efficiently and cheaper, integration of solvent-based CO_2_ capture and conversion. We present the fundamentals and benefits of integration within a changing energy landscape (*i.e.*, CO_2_ from point source emissions transitioning to CO_2_ from the atmosphere), and how integration could lead to lower costs and higher efficiency, but more importantly how CO_2_ altered in solution can offer new reactive pathways to produce products that cannot be made today. We discuss how solvents are the key to integration, and how solvents can adapt to differing needs for capture, conversion and mineralisation in the near, intermediate and long term. We close with a brief outlook of this emerging field of study, and identify critical needs to achieve success, including establishing a green-premium for fuels, chemicals, and materials produced in this manner.

## Abstract 2

In this perspective, we detail how solvent-based carbon capture integrated with conversion can be an important element in a net-zero emission economy.

## Graphical abstract

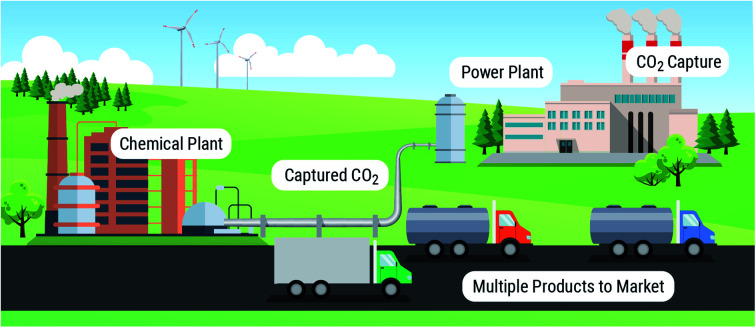

## Introduction

As the world continues to drive toward a net zero economy, fossil fuels will still be needed to supplement renewables during this transition to net-zero emissions. In this context, it is generally understood that CO_2_ capture and storage (CCS) technology has the potential to play a significant role in meeting climate targets.^1,2^ Presently, the biggest barrier to carbon capture technologies is the high total cost of capture, which comprises a combination of both energy demand and capital costs. In the past 20 years, there have been great gains in energy efficiency, though reductions in CAPEX remain challenging due to limited number of commercial systems in operation. To date, the total costs of capture are cheapest for concentrated gas streams such as coal-derived flue gas or cement kilns, spanning $47–80 (USD) per tonne CO_2_.^3^ Notably, there are escalating costs from capturing CO_2_ as the concentration becomes increasingly dilute from natural gas combined cycle (NGCC) flue gas ($80–100 (USD) per tonne CO_2_)^4^ and even higher costs and complexity for highly dilute streams, *e.g.* negative emission technologies (NETs) such as direct air capture (DAC) costs $150–1000 (USD) per tonne CO_2_.^5,6^

Solvent-based processes are the most mature CCS technologies owing to their significant commercial deployment for the purification of natural gas.^7^ While not the lowest energy carbon capture and separation (CCS) approach, solvents can achieve 60% thermodynamic efficiency, while having the lowest total costs of capture ($45–47.1 (USD) per tonne CO_2_).^8^ Most importantly solvents are the only technologies that can currently be manufactured at the requisite scale^9^ for point-sources such as coal and natural gas powerplants, cement kilns and steel furnaces.

In the past decade, solvent-based processes have undergone numerous changes in formulation and process to increase efficiency while reducing costs for point-source emissions. The field has seen a shift from simple strippers to more efficient configurations such as, lean-vapor compression with absorber intercooling or two-stage flash regeneration as a means to recoup heat, whereas the latter also is much cheaper and also bypasses the energy-intensive first stage of CO_2_ compression.^4,10–14^ Similarly, solvent formulations have shifted from first-generation aqueous alkanolamines to more complex second-generation aqueous amines, and now into third-generation water-lean solvents that are faster at absorbing CO_2_ and are more energy efficient to regenerate.^7,15^ Ultimately, leading solvent technologies that project to be 19% cheaper than Shell's CANSOLV technology.^16^

While attractive from a cost, ease of manufacture, and timeliness perspective, solvent-based processes should not be considered a panacea for CCS. This is because solvents have been primarily designed to mitigate point-source emissions but not those of legacy emissions using Negative Emission Technologies (NETs). In the near term, it may make sense to focus CCU applications on fixed point sources, thus contributing to the near-term avoidance of emissions, and the development improved capture technologies. In the longer term, however, it will be important to obtain non-lithospheric carbon as an industrial feedstock, and in this context, the further development of direct air capture (DAC) technologies will be key. Solvents can, therefore, be considered the cornerstone of a foundation on which many carbon capture technologies will be built to achieve the global goal of deep decarbonisation. Thus, our focus should be primarily on deployment so we can initiate large-scale emission reductions, but also so subsequent technologies and later stage NET approaches can benefit from the CO_2_ transport and storage infrastructure that will be established in the process, as well as markets for CO_2_, and CO_2_-containing materials.

The driver to increased deployment of CCU today is either to introduce regulatory requirements or to provide industry an economic incentive to utilise captured CO_2_. Outside of a few markets of enhanced oil recovery (EOR) and niche CO_2_ use for agriculture, there are limited markets for CO_2_ at meaningful scales. Thus, one way to reverse this reality is to provide more economic incentives for CCU, which could come from one of or a combination of tax credits, such as a modification from the well-known 45Q credit, or policy drivers like buying “green” mandates. There are dozens of materials that can be made from CO_2_,^17^ and whilst their markets are small relative to the scales required for whole-economy net zero, there remains potential for a meaningful contribution.

Owing to their unique ability to both cost-effectively capture CO_2_ at industrial scale and act as a medium for chemical conversion, solvent-based processes are the prime medium for integrated capture and conversion. Starting with the 2019 paper from the National Academy of Sciences on transforming separation science,^18^ there have been reviews, workshops, and Faraday transactions that thought leaders have begun to set the stage for reactive separations related to CCU.^2,19–21^ We build on these initial efforts and thoughts, and offer a more detailed definition of integrated capture and conversion, and identifying what is needed to make it more tangible. In this contribution, we identify the needs and priorities for capture and conversion in solvent-based processes in the *near*, *intermediate*, and *long-term* focusing on how solvents can lay the foundation on which timely and potentially profitable deep decarbonization can be built.

## Discussion

### Nature's integrated capture and conversion

Over millennia, natural systems have had time to perfect integrated capture and conversion of gas molecules. There are a handful of natural examples of systems (*e.g.* hemoglobin, carboxylic anhydrase) that capture and chemically convert atmospheric gases. Here, for completeness, we briefly describe examples that mirror our carbon-based energy systems.

We first highlight aerobic respiration, which involves O_2_ being absorbed, activated, and then transported to be used as a chemical oxidant. Respiration is analogous to the combustion of fossil fuels to release energy. O_2_ is absorbed in the lungs, being chemically coordinated to Fe^2+^ in heme groups found at the active site hemoglobin in red blood cells. This resulting iron superoxide (captured and activated O_2_) is circulated in the blood stream to muscle cells where O_2_ is consumed during chemical oxidation of fuel. This reaction provides energy for locomotion and heat. Unlike our combustion of fuels, in respiration, after O_2_ is consumed, the chemical process continues in reverse, removing 20–25% of the body's waste CO_2_*via* coordinated transport by the same heme group in hemoglobin. The waste CO_2_ is expunged by the lungs during exhalation, in a continuous process of inhalation and exhalation.

Plants capture, activate, and then convert CO_2_ into glucose in the Calvin cycle. CO_2_ is first absorbed in the chloroplasts, being first acted upon by the enzyme ribulose-1,5-bisphosphate carboxylase-oxygenase (RuBisCO). In RuBisCo, the active site includes the Lysine moiety which chemically fixates CO_2_ as a carbamate (captured CO_2_) that is stabilized by a Mg^2+^ in the active site. The captured/activated CO_2_ is then transcarboxylated to ribulose 1,5, biphosphate, making two molecules of glycerate-3-phosphate, which then are converted to glucose in later stages of the Calvin cycle.

Humans also perform capture and conversion of CO_2_ in metabolic pathways such as gluconeogenesis, fatty acid synthesis, and amino acid catabolism. In our bodies, CO_2_ is first hydrolyzed by the enzyme carbonic anhydrase, making a bicarbonate which is first transcarboxylated to the biotin co-factor with the aid of adenosine triphosphate by any of the four carboxylase enzymes. Once the CO_2_-biotin carboxylate adduct is formed, the activated CO_2_ is subsequently reacted with metabolites such as pyruvate to make fuels such as glucose and fatty acids. Conversely, extremophiles such as cyanobacteria achieve glycogenesis by means of a slightly different approach due to the absence of light to provide the energy source. In the deep ocean, bacteria capture CO_2_ and H_2_S from deep-sea vents, and produce glucose by using H_2_S as a chemical reductant to reduce CO_2_ captured as a carbamate.^22,23^ This chemotrophic capture and conversion of CO_2_ is driven by the deep-sea vents, because of the immense heat providing the driving force for the chemical reactions.

**Fig. 1:**
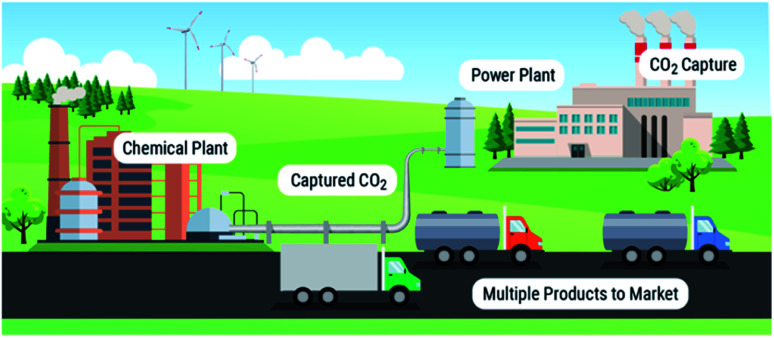
Our vision of a 21st-century point source manufacturing center from CO_2_.

We point to these systems as examples, as there are already energy technologies that emulate these biological processes. Aerobic respiration can be considered an analogue for chemical looping combustion, and glucogenesis by organisms such as algae is the fundamental process behind algal biofuel production. As these types of technologies exist for performing chemical energy storage and release of chemical energy during combustion, we can see a possibility/reality where solvent-based carbon capture and conversion are integrated to emulate natural systems. From this concept, we can envision a modular multi-product chemical manufacturing facility (Fig. 1) similar to Shell's Pearl GTL plant,^24^ albeit making value-added products from CO_2_ instead of natural gas.

### Why integrate capture and conversion?

While many reactions of CO_2_ have been reported in the literature, there is an unwritten assumption that CO_2_ is free. In reality, CO_2_ typically has to be recovered from a range of sources of varying dilution, and each of the CO_2_ absorption, desorption, compression, and transportation unit operations come at a cost. Reactive separation of CO_2_*via* conversion in the capture solvent is a logical approach to reduce the cost and energy demands requisite for CO_2_ capture. Natural systems are integrated because it makes sense from an energy perspective, though there are other potential benefits of doing so. Integration also provides improved thermodynamic efficiency, the ability to alter or bypass limiting chemical equilibria, the use of new drivers to increase the chemical potential for separation and conversions (much like reactive distillations), and most importantly, more favorable economics due to reduced redundancy. We further expand on potential drivers, detailing how each can be a motivator for integration.

### Integration can be used to enhance CO_2_'s reactivity for conversion

As we aim to process (hopefully) many millions of tonnes of CO_2_ a year, the energy demand and rates greatly influence the CAPEX and OPEX of any utilization approach, and any means to reduce either, will improve the prospects of commercial viability. To date, approaches to utilize CO_2_ have focused on optimizing and refining every variable available except the most obvious one, the CO_2_ itself.

**Fig. 2:**
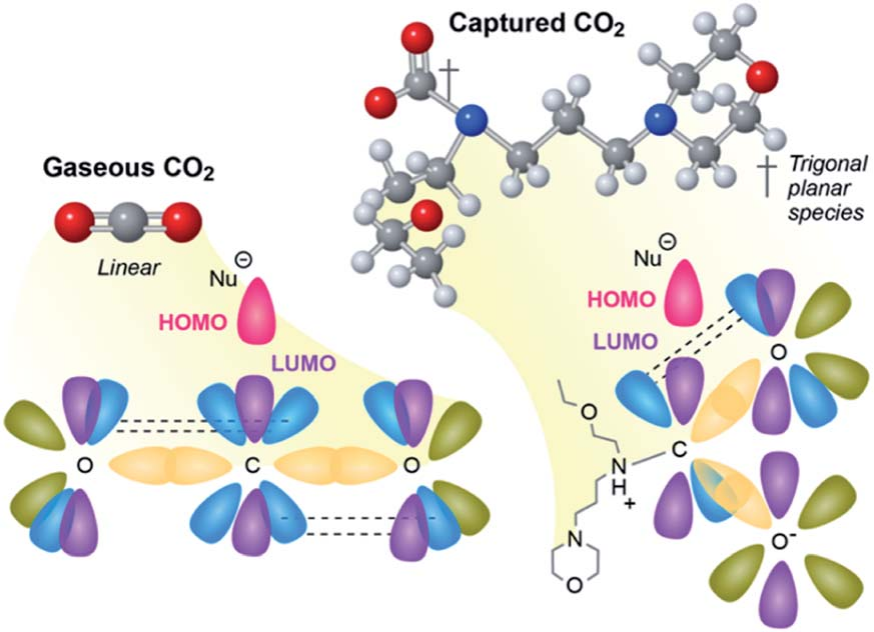
On orbitals and nucleophile attack/availability.

CO_2_ is a very stable molecule, thermodynamically and kinetically speaking. Revisiting Valence Shell Electron Pair Repulsion (VSEPR) theory, CO_2_ (gas) is sp hybridized, and strong overlap of the bonding orbitals limits reactivity. Reactions that involve the central carbon (O=C*=O) require a nucleophile to deposit paired electrons in a small and highly shielded sp antibonding orbital (Fig. 2, left). As a result, the activation energy (*E*_act_) for most reactions of CO_2_ are high, and, consequently, reaction rates are slow. This high activation energy is why reductions of CO_2_ in its native state require catalysts that function at high temperatures (>300 °C) to achieve an appreciable rate.

Conversely, chemically captured CO_2_ is trigonal planar sp^2^ hybridized anionic carboxylates. This hybridization is nearly universal in solution (*e.g.* as bicarbonate in sea water, or carbamates in aqueous amines) and in amine-functionalized solid sorbents that capture CO_2_ as carbamates. This molecular geometry is sterically and electronically more favorable for nucleophilic attack because the p* antibonding orbital is perpendicular to the plane of the molecule (Fig. 2, right), making it less shielded and more accessible. Further, when CO_2_ is reduced or attacked by a nucleophile, the resulting CO_2_-containing intermediate or species are commonly trigonal planar (sp^2^). This is in part why the majority of the natural systems, *e.g.*, RuBisCO, described above capture, transport, or react CO_2_ in an activated sp^2^ state (*e.g.*, biotin-carboxylates, bicarbonate).

Anionic carboxylates are negatively charged, but they *can* be reduced in catalytic processes. Anionic carboxylates readily coordinate to cationic metal complexes and heterogeneous catalysts. Once the carboxylates are coordinated to metals, they become charge-neutral, enabling reduction. It has recently been demonstrated that alkylcarbonates are more reactive toward ruthenium hydride complexes than gas-phase CO_2_ under comparable conditions *via* an inner-sphere reduction.^25^ It is likely that carbamates share a similar reactivity. We have also shown coordination of CO_2_-containing ions to heterogeneous catalyst interfaces at temperatures comparable to CO_2_ release from solvents (∼120 °C),^26^ suggesting durable heterogeneous catalysts could be employed for integrated capture and conversion approaches. For the recent reviews related to integrated capture and conversion, see ref. 27–29.

### The capture solvent can aid conversion

A benefit of performing a conversion reaction *inside* a carbon capture solvent allows us to proceed at ambient pressures of CO_2_. In most reported catalytic hydrogenations or conversions, higher pressures are applied to provide a high concentration of CO_2_ in solution. For example, the mole fraction of physically dissolved CO_2_ in water and organics are ∼0.00044 and >0.01, respectively, at 40 °C under 1 atm CO_2_.^30–35^ In particular, CO_2_ pressures of 15–30 bar CO_2_ are common concentrations for conversions because CO_2_ is a non-polar molecule whereas the conventional solvents used are polar. Conversely, as the CO_2_ gas is already chemically reacted with the capture solvent, high CO_2_ pressure is not needed when the conversions are performed in a capture solvent. The CO_2_-loaded carbon capture solvents (after treating a high [CO_2_] flue gas) entail CO_2_-rich loadings of approximately 5 wt%.^4,36^ From a systems perspective, the ability to react at lower pressure negates the cost and duty of CO_2_ compressors to achieve sufficient concentrations of CO_2_ in solution.

The rate of chemical reactions can be greatly influenced by solvent effects. Chemically selective solvent-based carbon capture is a reaction between a weak acid (CO_2_) and a strong base (amines). As like all acid–base reactions, proton transfer and charge solvation are the primary reaction steps, which are favored in polar protic or polar aprotic solvents such as water, propylene carbonate, glycols, sulfolane, and, more recently, ILs.^37,38^ Solvents in general, favor the reaction because they are able to provide charge solvation and facilitate proton transfers. Solvents are employed in many organic chemistry reactions and thermocatalytic conversions because they solvate catalysts in addition to stabilizing high-energy transition states (*i.e.*, rehybridization of CO_2_), in turn lowering the activation energy of a reaction (*E*_act_) few of kcal mol^−1^,^39^ and thereby allowing reactions to proceed more readily. As carbon capture solvents are polar protic, or polar aprotic media, these solvent effects provide a lever to control reactivity that remains unavailable to gas-phase catalytic processes.

### Changing speciation allows for bypassing limiting chemical equilibria, enabling lower T and P reactions

The reactions of CO_2_ in gas phase are very different from the condensed-phase chemistry. For instance, in the conventional gas-phase CO_2_ hydrogenation to methanol reaction, the typical reaction intermediates are formate (HCOO_ad_), acetal (OCH_2_O_ad_), and methoxy (OCH_3ad_),^40,41^ whereas in the condensed phase, *inside* a carbon capture solvent, the intermediates involved are carbamate (–NCOO^−^) or carbonate (–OCOO^−^), formate (HCOO^−^), and formamide (–NCHO)/formate ester (–OCHO).^25,26,42–46^ Depending on the catalyst, solvent, and reaction conditions (T, P, diluent, additives *etc.*), we can selectively form one of these value-added chemicals/intermediates—formate (or formic acid), methanol, and methane.

The CO_2_ hydrogenation to formic acid is strongly endergonic 
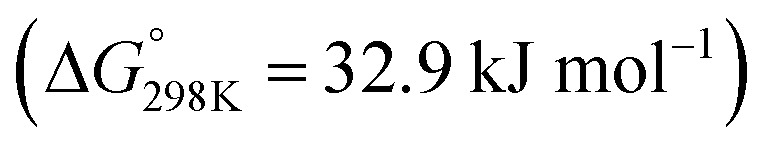
. However, in the presence of an ionic liquid or organic/inorganic bases, the reaction becomes exergonic, moving equilibrium toward formic acid and formate.^39^ A base is typically used to drive the formation of formate upon CO_2_ hydrogenation. Then, the formic acid is separated from the base by ion exchange or thermal cleavage. Coincidentally, the CO_2_ capture solvents typically contain amine units, which can act as a base to promote the formation of ammonium formate. The hydrogenation of captured CO_2_ to formate has been shown by us and others in both aqueous and water-lean solvents.^25,42,45,47,48^

Low-temperature methanol synthesis from CO_2_ has been gaining lot of attention recently.^26,27,43,49–52^ The methanol formation from CO_2_/H_2_ is an exothermic process, and, based on the entropy, low temperature will give higher conversion to methanol, but due to slower reaction kinetics, the methanol synthesis is typically performed at high pressures and temperatures (>200 °C). The presence of alcohols and bases were shown to favor the formation of methanol at low temperatures (<170 °C) by reacting with formate to make formamides and formate esters to drive reaction in solution phase.

C_1_ products like methanol or methane made from the hydrogenation of CO_2_ generates quantitative amounts of water as a byproduct. The use of low-water or water-lean capture solvent is an attractive option compared to aqueous solvents because the excess water in aqueous solvent can reverse the reaction. The use of a water-lean capture solvent also offers higher concentrations of dissolved CO_2_ as compared to aqueous solvents, lowering the temperature and pressures required for synthesis, thereby significantly suppressing the reverse water gas shift (CO_2_ + H_2_ → CO + H_2_O).

### Heat from conversion can be recovered to drive regeneration of the solvent or reused elsewhere

Solvent-based carbon capture is an exothermic reaction, driven by the large enthalpic driver between the heat of protonation from (most commonly, but not limited to) carbamic acid (made from amines and CO_2_) and the amine. Mathias has identified an optimal heat of solution for capture of CO_2_ from point sources to span between −60 to 85 kJ mol^−1^, where the thermodynamics are optimal for the separation from coal-derived flue gases.^36,53,54^ The regeneration of the solvent (release of CO_2_) is endothermic, requiring an equally strong if not stronger enthalpic driver to force the regeneration. Solvents have been regenerated by many approaches, including but not limited to electrochemical, pH, or thermal means, with the latter being the most common.^7,36,55–58^ In each case, reactivation of the solvent requires a substantive amount of energy (2.0–3.3 GJ per tonne CO_2_) to reactivate the sorbent and deliver a relatively pure CO_2_ stream.

Conversely, most value-added products that could be made from CO_2_ are made from high-energy reagents like metal oxides, silicates, epoxides, or reductants such as e^−^ or H_2_. The most common conversion of CO_2_ is reduction to chemicals or fuels such as formate (or formic acid), formamides, methanol, or methane, all of which are downhill energetically due to strong exotherm driven by the production of water as a byproduct in most of these cases. These most common products made from reduced CO_2_, entail reaction enthalpies of −84 kJ mol^−1^ (ammonium formate), 33 kJ mol^−1^ (dimethyl formamide), −49 kJ mol^−1^ (methanol), and −165 kJ mol^−1^ (methane). Similarly, mineralisation of CO_2_ is highly exothermic, with enthalpies for mineralisation of −118 kJ mol^−1^ and −179 kJ mol^−1^ for MgCO_3_ and CaCO_3_ respectively.

**Fig. 3:**
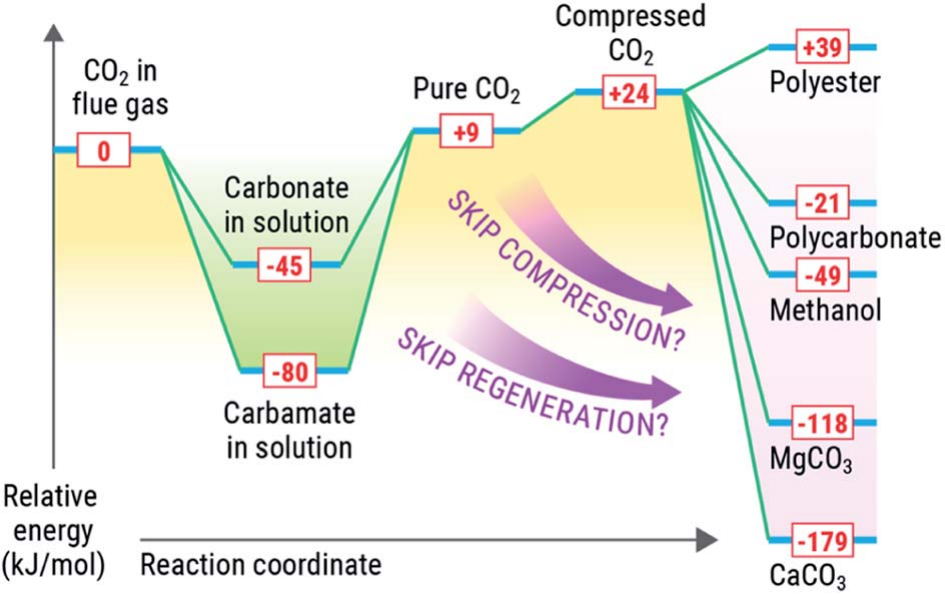
Conceptual energy comparison for capture and conversion of CO_2_.

It is desirable to optimize thermodynamics of *both* (capture and conversion) processes to minimize energy losses. Integration of the capture and conversion allows this to happen because the scale of the endotherm in regeneration of the solvent is comparable to the exothermic conversion. Thus, from a systems perspective, depending on the availability and quality of the heat of the conversion reaction, we could and should aim to offset the regeneration of the solvent (Fig. 3) or repurpose the heat elsewhere in an integrated CO_2_ to chemicals plant. We concede that there is a large difference in scale between conversion and capture, in that there is not enough reagent to consume all captured CO_2_, and high-energy reagents like minerals and H_2_ require energy-intensive processes to produce. We posit that integration could be viable for slipstreams that can scaled to the availability of reagents and quality of heat to drive the solvent regeneration (or other processes) *via* chemical conversion.

### Removing units of operation reduces energy and capital costs

As mentioned previously, a capture process has an absorber, heat exchangers, pumps, and heaters, all of which will be in a conversion process as well. Also, from a capital perspective, utilizing systems and reagents for multiple steps removes the redundancy, enabling a cheaper integrated system as compared to disparate systems. Similarly, if a smaller, modular capture and conversion unit were made, the system could be configured without a compressor, potentially saving money and energy in the process.

Also, from a systems perspective, converting CO_2_ into condensed-phase products saves energy because this approach can bypasses the need for the high-compression energy to compress CO_2_ to a supercritical state for transportation in pipelines. In solvent-based processes, CO_2_ is released from a solvent at ∼1.8 bar at 120 °C, though more recent approaches by Rochelle have shown sizable energy savings if CO_2_ is thermally compressed and released at 6 bar, which allows solvents like piperazine (PZ) to bypass the first (and most energy-intensive) stage of compression.^13,59^ The compression energy of CO_2_ according to the US DOE Case 12B baseline is about 32 MW,^4^ not counting the energies associated with transportation, which of course are variable. If the slipstream conversion can make CO_2_ into a product that exists in the condensed phase, this energy demand could be eliminated for compressor work, potentially reducing its size and cost.

### Selling CO_2_-derived products can pay for the initial separation

CCS has been deployed in limited cases such as (Port Charles, Aquistore BD3) because there are limited market incentives to cover the costs of capture ≥$47.1 (USD) per tonne CO_2_ and $20 (USD) for transportation and storage. Selling CO_2_-derived products could off-set some costs associated with carbon capture, thereby providing enough incentive to encourage commercialization.

Presently, there are a few target large-volume economically profitable and scalable chemicals that could achieve approximately 0.3 to 0.6 Gt CO_2_ per year reductions by the year 2050, with breakeven costs of ∼$80 to $320 (USD) per tonne of CO_2_.^60^ Urea (140 Mt CO_2_ per year breakeven at $100 (USD) per tonne), and polycarbonate polyols (10–50 Mt per year breakeven at $2600 (USD) per year) could be initial targets. It is noteworthy that, under the revisions proposed to the 45Q tax credit, incentives to capture and geologically sequester CO_2_ were proposed in the range of $85–200 (USD) per tonne – not dissimilar to the range required to make some CO_2_ capture and conversion projects economically viable. We further observe that these and other economic assessments assume decoupled capture and conversion, with the conversion proceeding with on a nearly pure CO_2_ stream. Integrated capture and conversion systems could reduce energy demands, and selling prices, potentially lowering breakeven points for urea, polyols, and potentially fuels. Further, breakeven points could become lower for other chemicals that are not presently profitable or scalable, giving us a larger market for CO_2_-containing products and thus a larger potential to avoid greenhouse gas emissions. It will, however, be important to recognise that the fate of this CO_2_ will be its emission to atmosphere, and in a net zero paradigm, these emissions must be accounted for and compensated by the permanent removal of carbon dioxide removal from the atmosphere, such as bioenergy with CCS (BECCS), DAC, enhanced weathering (EW) or another equivalent pathway. Thus, in the longer term, CO_2_ used in the production of fuels, and platform chemicals will need to come from the atmosphere, or other equivalently sustainable source.

### Bringing it all together, an integrated capture and conversion example

Combining all of the advantages described earlier, we recently showed economic and energetic benefits of an integrated CO_2_ capture and conversion process to make CO_2_-neutral synthetic natural gas. In this process, the CO_2_ is first captured using a single-component water-lean solvent, 2-EEMPA (total costs of capture at $47.1 (USD) per tonne CO_2_).^8,61^ The CO_2_-rich solvent is mixed with H_2_ and hydrogenated in the presence of a commercial Ru/Al_2_O_3_ catalyst at 120 °C which is ∼3 °C higher than the solvent's reported regeneration temperature. The energies associated with CO_2_ compression are lessened as the reaction is performed in the condensed phase, and similarly the exothermic hydrogenation is used to partially offset the solvent regeneration enthalpy. In this process >90% of the CO_2_ is hydrogenated, producing a mixture of hydrocarbons, mostly methane at temperatures which are less than half of conventional gas-phase Sabatier reactions. The technoeconomic assessment (TEA) of the integrated process from CO_2_ captured from a coal-fired powerplant reported a reduction in the capital cost and minimum synthetic natural gas selling price by 32% and 12%, respectively.^61^ Further, the integration of the two processes enabled an increase in thermal efficiency by 5% as compared to separate capture and conversion. The improvement in thermal efficiency, reduction in capital costs and product selling price (compared to conventional non-integrated process) all point to integration as a key driver that could provide comparable benefits to other CCU systems.

### Where should integrated solvent-based CCUS focus?

With the case laid out for integrated capture and conversion, we first note that there are three time periods of impact, each with differing needs. Here we highlight areas of need with respect to chemistry, catalysts, processes, and markets for each of these time periods.

### In the near term, finding a way to further incentivize the separation is critical

Improvements in CO_2_ capture technologies in the near term (<10 years) are needed to enable the energy sector to deploy carbon capture on point-source generators (power plants). Thus, near-term efforts should focus on integrated conversion of CO_2_ into a range of value-added products, that can further develop CCU at meaningful scales and establish a marketplace for CO_2_-containing materials.

Here research approaches should assess the viability of integration of capture and conversion of CO_2_ into economical and scalable chemicals like urea and polyols, and carbon-neutral energy-carriers such as methanol, methane, formic acid and dimethylether, similarly to what has been shown for methane.^61^ Combined theoretical and experimental R&D efforts should assess coupled chemical processes as a means to conjoin two chemical processes thereby bypassing the energies associated with regeneration of the capture medium and compression of CO_2_ (Fig. 1). These integrated reactive separation approaches allow us to design new condensed-phase catalytic systems that bypass limiting chemical equilibria of conventional high temperature gas-phase reductions of CO_2_ into energy-carriers.

*True integration of capture and conversion requires a solvent that can perform both capture and conversion.* There are many notable groups doing work on thermocatalytic conversions of CO_2_ providing insight on the basic reactivity, though we would define these “amine-promoted” conversions of CO_2_ as they use additives that are not carbon capture solvents.^26,43–45,47,62–64^ All of these approaches utilize gas-phase catalysis, or the use of chemicals or promoters that have not yet been validated as viable solvents for CO_2_ capture. Polyamines, volatile secondary or tertiary amines or solvents and promoters like tetrahydrofuran or ethanol are either too viscous, too energy inefficient, or too volatile to be used in capture technologies.

**Fig. 4:**
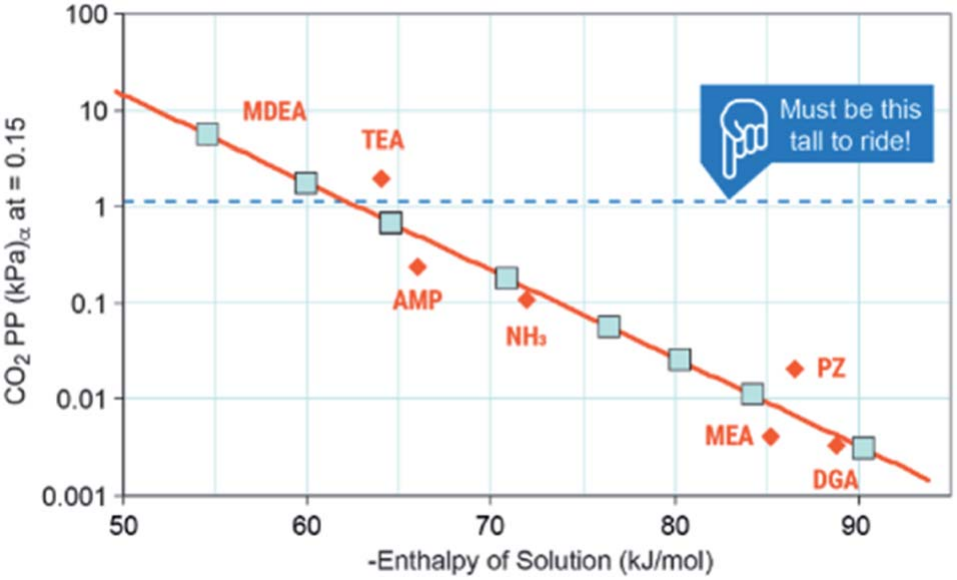
Solvent ranges of viability. Image adapted with data from Mathias, *Int. J. Greenh. Gas Control*, 2013, **19**, 262–270.^53^

The first criteria for a viable capture and conversion solvent should be the capability of absorbing a sufficient amount of CO_2_ from a stream such as coal-derived flue gas or a cement kiln.^4^ The maximum uptake under equilibrium that any solvent could achieve is the equilibrium partial pressure of CO_2_ (P*) over the liquid at a given temperature. As post-combustion processes absorb at approximately 40 °C, they are configured to achieve >90% capture of 1.4 kPa CO_2_, the P* needs to be ∼1.4 kPa to perform the separation. Mathias's work provides critical information on the thermodynamics of capture (Fig. 4).^53^ He identifies that the minimum enthalpy of solution that could perform this separation is ∼−60 kJ mol^−1^ with a favored “Goldie Locks” range of −65 to 85 kJ mol^−1^. This range clearly shows why viable post-combustion solvents are chemical sorbents because physical solvents such as Rectisol and Selexol are too weak to capture 90% CO_2_ at 40 °C. We would also like to point out that if a solvent cannot work for concentrated streams like coal or natural gas exhaust, conditions in DAC are significantly more challenging because the P* of CO_2_ in air is a meager 0.04 kPa, meaning solvents need to be even stronger or have other drivers (*e.g.* precipitation) to capture CO_2_ any meaningful amount of CO_2_. Thus, one area of need is for more data on the P* of common solvents or promoters used in CO_2_ conversion to identify which, if any, of these co-solvents or promoters are capable of the initial separation.

The second criteria is that the solvent or co-solvent cannot be volatile or viscous. As we have detailed before, solvents are designed to minimize evaporative losses in the absorption side of the process.^36^ Coal-derived power plants and other exhaust streams flow millions of pounds of gas an hour over a liquid in the absorber, meaning any volatile solvent such as triethyl amine or ethanol or co-solvent like THF will evaporate in the absorber column. Second, the rheological properties of the solvent need to be considered, notably that viscous ionic liquids or polymeric amines are prone to become quite viscous after CO_2_ complexation, which will cause greatly reduced rates of mass transfer, increasing the size and cost of the absorption unit.

Last, integrating capture and conversion requires a minimum level of TEA against a plausible reference case, such as those set by the U.S. Department of Energy's cost and performance baseline or comparable targets. Industrial benchmarks like 5 M monoethanolamine (MEA) or Shell's Cansolv have reboiler duties of 3.3 and 2.4–2.5 GJ per tonne CO_2_ and projected costs below $66 and $50 (USD) per tonne respectively.^4^ If a solvent used for capture does not meet or exceed these benchmarks, then they should not be considered targets in an integrated approach. We should instead focus on chemical conversions in validated post-combustion solvents.

There are only a few integrated capture and conversion approaches using what we define as “viable” post-combustion carbon capture solvents. Thermocatalytically, we have shown that CO_2_ could be made into methane in a single-component water-lean solvent 2-EEMPA.^61^ Similarly, Leitner and Francio have shown hydrogenation of CO_2_ hydrogenation using a 20 wt% MEA solution and homogenous catalyst in biphasic systems.^65^ Conversely, Sargent has shown an elegant electrocatalytic approach to convert CO_2_ into syngas in 5 M MEA, a process that was a finalist for the carbon XPRIZE.^66^ We have also shown the ability to coproduce two value-added chemicals—propylene glycol and methanol—at the same time with no waste and 100% atom efficiency, helping us utilize all the CO_2_ and avoid producing waste.^50^

### In the intermediate term we need to expand solvents to capture CO_2_ from a greater number of point sources and reconstitute CO_2_ into materials or products that are CO_2_-negative

CCUS in the intermediate term (10–20 years), we assume continued growth of renewable energy to meet global energy needs, though there will still be some enduring point sources such as coal-, natural gas-, and biomass-fired power plants, as well as cement kilns, and steel plants that will continue to emit concentrated CO_2_ streams. Here, solvent-based capture will first need to be adapted for treatment of these remaining concentrated exhaust sources.

Similar to the near term, utilization will require continued monetization of CO_2_, which is critical to achieve widespread deployment of CCU technology. Here, it makes little sense to continue to make C_1_ products identified in the near term, as those markets should already be established, and those products are CO_2_-neutral at best. To achieve tangible NETs, we will need new integrated CCU technologies that can produce a multitude of large-volume CO_2_-containing chemicals and materials that do not emit CO_2_ (*e.g.*, polymers, composites). When we define NET's, we abide by the guidelines set forth by Ramirez.^67^ These materials represent sizable markets and potential CO_2_ sinks, though the valorization can be further driven by tax incentives. One example, the revised 45Q tax credit in the US, which provides $85 (USD) per tonne if the CO_2_ is “permanently sequestered”, noting that further clarification on the definition of “permanent” in this context will be required.

*CO*_*2*_ *utilization must follow the principles of green chemistry and be atom and energy efficient*.^2,68,69^ This means, that our products need to contain the entire CO_2_ molecule, not fragments or materials that contain 1–3 atoms of the CO_2_. Similarly, we should avoid products and reactions that require high-energy C–O bond breakage because that adds even more energy demands to CCUS approaches.

Presently, there are a handful of chemicals that could utilize CO_2_ in its entirety. There have been many reviews that cover the size and scale of available markets though there is a disconnect in the commercial viability of the products we can presently make that consume the entire CO_2_ molecule. Reactions that utilize the entire CO_2_ molecule include carboxylation, cyclization, and polymerization with epoxides. CO_2_ polymerizations to make poly and cyclic carbonates, and reductions into fuels involve electrophilic attack of the central carbon in CO_2_ by strong nucleophiles. There are several approaches to make cyclic and polymeric carbonates from gaseous CO_2_, though there are only a few examples of integrated capture and conversion to materials.^70^ We laud the pioneering groups (Inoue, Darrensbourgh, Coates, and others)^71,72^ who are leading the field, but the desirable polycarbonates cannot yet be made with the requisite polydispersity index and molecular weight at a low enough cost. Further, polycarbonates made from CO_2_ have inadequate mechanical strength and low glass transition temperatures and are prone to thermal degradation and hydrolysis (releasing CO_2_ after a few years), thus limiting the application scope.

We suggest the focus in the long term should be attempting to make chemically durable, large-volume commodity materials that contain CO_2_ equivalents in their linkage that cannot yet be made from CO_2_. Viable targets include but are not limited to polyurethanes (NC(O)O) and polyesters (OC(O)O). Polyurethanes are valuable materials in adhesives, coatings, and foam insulation, with millions of tons a year produced and market sizes in the billions of USD.^73^ Similarly, polyesters are used ubiquitously as bottles for the beverage industry, and fabrics for the textiles industry with millions of tons a year and market sizes billions of dollars per year.^74,75^ Industrially, polyurethanes are made by polyaddition between different diisocyanates and diols (or polyols). The polyols used for the polyurethane synthesis can be polyether, polyester, polycarbonate, acrylic polyols, or polybutadiene polyols.^76^ The ability to use a wide range of polyols, isocyanates, and additives for polyurethane synthesis to achieve different properties makes them suitable for many applications. The polyether polycarbonate synthesized from epoxide and CO_2_ is used to make polyurethane flexible foams.^77,78^ It should be noted that these flexible foams containing up to 20% CO_2_ are already commercialized by the German polymer manufacturer Covestro and used in mattresses and upholstery furniture.^79^

On the other hand, isocyanates, the other raw material used for polyurethane synthesis, are synthesized by treating the corresponding amines with phosgene. Using CO_2_ as a C_1_ source for isocyanate synthesis instead of phosgene (which is highly toxic and prepared from CO and Cl_2_), is attractive from an economical and sustainability standpoint, but are still in exploratory stages.^80^ Alternatively, an isocyanate-free route to directly incorporate CO_2_ into polyurethane linkage in fewer steps is also in its infancy.^81^

Conversely, polyesters are conventionally made by a polycondensation reaction between dicarboxylic acids and diols or by ring opening polymerization (ROP) of lactones. Direct use of CO_2_ as a comonomer for copolymerization with diynes and terpolymerization with diynes and dihalides has also been explored in addition to indirect approaches where the CO_2_-derived polymerizable building blocks (mostly lactones) are used to produce polyesters. The direct polymerization of ethylene (or olefins) and CO_2_ is the straightforward approach to produce polyester, but it remains elusive mainly because of the high activation energy for the CO_2_ insertion into the growing polymer, the facile homopolymerization of ethylene competes with the alternating CO_2_/ethylene copolymerization.^82–85^

In the long term, the question is, can the captured CO_2_ inside the solvent reduce the free-energy barriers for desired reactions of CO_2_? Reducing our dependence on fossil resources will require an atom-economical, energy-efficient, cost-effective, industrially scalable, and environmentally friendly approach to utilize CO_2_ as a monomer in polyurethane and polyester synthesis. It should be noted that catalysts will also play a crucial role to achieve these transformations.

In addition, achieving near-term needs, methanol can be a CO_2_-sourced building block to produce various polymers. Conversion of methanol to olefins and subsequent polymerization or epoxidation can provide polyethylene, polypropylene, polyesters, polycarbonates, and others. This will allow us to incorporate most of the CO_2_ into polymers and to rely less on the fossil resources for hydrocarbons.

Our inability to make polyesters and polyurethanes is in part due to our antediluvian approach of converting CO_2_ either *via* addition and elimination reactions that rely on nucleophilic attack of the weakly electrophilic central carbon, or electrophilic attack on the weakly nucleophilic oxygens. Revisiting the orbital hybridization discussion above, if we are attempting to polymerize or co-polymerize CO_2_ in its linear SP hybridized state, we are limited in the types of reagents or catalysts that we can utilize. To date, the field has seen slow and steady improvements mostly driven by Edisonian research approaches. To date, the field has focused on altering catalysts, reagents, chelants, solvents, temperature, and pressure, but there is only so much that we can bend, distort, and chelate, with our current reagent pool. The lone variable left to be sufficiently altered is the CO_2_ itself. Captured CO_2_ are almost universally anionic sp^2^ hybridized carboxylates. This hybridization offers more electrophilic *and* nucleophilic active sites than sp hybridized CO_2_, so we question why we as a community should not assess reactivities of these different chemical species and bonds? For this reason, we should avoid being biased as we strive to develop CO_2_-containing materials. We should instead focus our efforts on the harder challenges of designing new reactivities that deviate from rudimentary approaches that rely on electrophilic and nucleophilic attack.

Similarly, catalysts will be an important element of this mid-term strategy, and they will need to be reinvented to operate in the condensed phase acting on captured CO_2_ in solution. Presently, the majority of catalytic approaches to convert CO_2_ operate on the assumption of outer-sphere reactions (reduction or transcarboxylation) where the CO_2_ does not directly (or is assumed not to) coordinate to the active metal. This greatly limits us as we strive to design new and more efficient catalysts. CO_2_ captured in a solvent will be in the form of anionic carboxylates which will directly coordinate to cationic metal sites, so our focus should be on designing catalysts or processes that operate *via inner-sphere* mechanisms in the condensed phase. Performing reactions in the capture solvent could be done at lower temperatures and pressures while also achieving the economic and energetic reasons identified above. Thus, our thinking on catalyst design and operation needs to align with (or unlock) the new reactive approaches for CO_2_ described earlier. Also note, the catalyst needs to be tolerant of the SOx and NOx in the flue gas.

Depending on the product of interest, the choice of the nature of the catalyst *i.e.* heterogenous or homogenous, used will also be critical, mostly from the separation standpoint. In the case of solid products such as polymers, homogenous catalysts will facilitate easy separation of the solid products from the catalyst and capture solvent. On the other hand, in the case of volatile liquid products such as methanol, the use the heterogenous catalyst will ease the separation of the catalyst from the liquid products and capture solvent. Then, the liquids products can be separated from the capture solvent *via* distillation. Similarly, in the case of gaseous products such as methane, the use of heterogenous catalyst will still work best for the separation of the product and capture solvent.

### In the longer term, solvent-based technologies will need to adapt to permanent NETs as the world prioritizes deep decarbonization

Long term (>20 years), large-scale carbon capture and utilization will have been de-risked, and commercialized, with the global markets for CO_2_ and its products will have been established.

There are many NETs identified as a part of a multipronged approach, including but not limited to enhanced weathering, seawater capture, afforestation, reforestation, and DAC, with the latter being the current trend in the CCUS community. The vision for DAC is the fabrication and deployment of millions of “artificial trees” powered by renewables to capture CO_2_ from air, coupled with CO_2_ utilization, though preferably sequestration. The inherent volatility of solvents and high energy demand for thermal-swing regeneration make solvents less attractive for integrated CO_2_ capture and conversion from dilute streams as in DAC. Similarly, afforestation and reforestation approaches are not amenable to integration with solvent-based capture processes. Solvents are unlikely to provide value to sub-surface sequestration as this step involves pumping almost pure CO_2_ into reservoirs.

*Solvents could, however, provide the means to expedite the glacial rate of mineral carbonation.* Mineral carbonation, *i.e.*, mineralisation of gaseous CO_2_ occurs with alkaline earth metals (*e.g.*, Ca, Mg), that are present in naturally occurring silicate and oxide minerals. There is a plethora of data on the viability, cost, and economics of the millions of metric tonnes of global mine tailings that could mineralize gigatons of CO_2_.^86–91^ Mineralisation is a spontaneous process that is downhill energetically,^92^ though mineralisation is not a panacea. While favorable enthalpically, the high kinetic barriers of these mineralisation reactions limit the rate of carbonation to a glacial pace. This is in part due to the mineralisation being impeded by the formation of passivating surface layers of carbonate.^90^

Recent efforts have focused on means to promote enhanced weathering (mineralisation), by mechanical, thermal, and chemical means. Mineralisation reactions are greatly influenced by surface area of the mineral that is reacting with the gaseous CO_2_. Currently, the easiest (and most inefficient) way to increase the rate of mineralisation is mechanical grinding of the rock to increase its available surface area. Second, the rate of mineralisation can be enhanced with elevated temperature and pressure, a process which also requires additional energy input. Third, mineralisation is best performed in aqueous solution, with recent sequestration studies showing that mineralisation is greatly enhanced by carbonating hot water.^93^ Mineralisation can be further expedited by the addition of chemical additives such as acids and bases to digest the passivating carbonate interface.

Solvent-enhanced weathering is a textbook example of an integrated capture and conversion approach. The primary benefit of integration is the exploitation of the strong exotherm of mineralisation (−118 kJ mol^−1^ to −179 kJ mol^−1^ for MgCO_3_ and CaCO_3_ respectively) to provide the regeneration enthalpy of the solvent (<85 kJ mol^−1^).^92,94^ Additionally, solvents can serve as a transport medium, providing a highly concentrated CO_2_ source to promote mineralisation. Solvents also consist of water and bases that are known to enhance the rates of mineralisation by digesting the passivating carbonate interfaces.

**Fig. 5:**
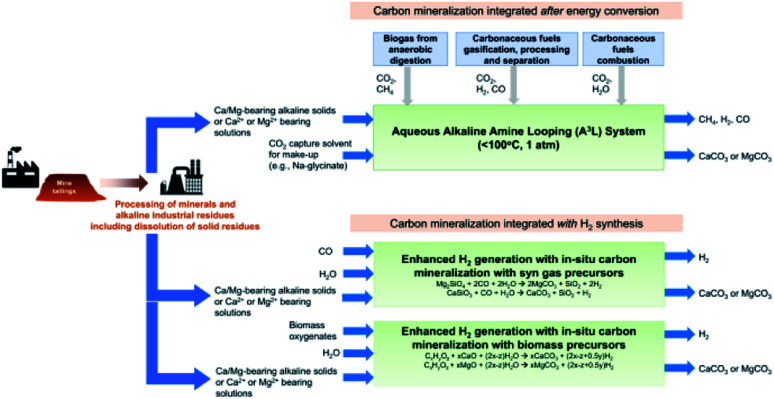
Conceptual solvent-based capture and concurrent mineralisation. Image from Gadikota *et al.*, 2021.^100^

While this may sound like fiction, this concept has already been demonstrated at the laboratory scale. We highlight the work of the groups of Gadikota, Liu, Park, and Bourgeois, showing the viability and improved performance of integration of solvent-based capture and mineralisation approaches (Fig. 5).^94–98^ Gadikota recently showed the effectiveness of MEA, sodium glycinate (NaGly), 2-amino-2-methylpropanol (AMP), and water-lean solvents such as 1,8-diazabicyclo[5.4.0]undec-7-ene (DBU), on the carbon mineralisation of CaO, CaSiO_3_, and MgO.^99^ Liu demonstrated the viability of capture using CO_2_-rich solutions of MEA, PZ, diethanolamine (DEA), AMP, *N*-methyldiethanolamine (MDEA) to perform subsequent mineralisation and precipitation of calcium carbonate as a means to regenerate the solvents.^96^ Bourgeois and Leclaire expanded integrated solvent-enhanced mineralisation to magnesium silicates, demonstrating enhanced rate and yield of mineral carbonation of MgSiO_4_ using aqueous solutions with polyamines.^97^ These works are of note because they further demonstrate that solvents can enhance the rate and degree of sequestration by mineralisation with silicates, and additional economic incentives by selling MgCO_3_, SiO aggregates, and valuable ores recovered from these processes. Integrated approaches like these demonstrate the feasibility and sizable befits of integrated capture and mineralisation.

With benefits come challenges. Integrated solvent-based capture and utilization will require significant R&D efforts to ensure long-term viability of this type of approach. The first challenge will be ensuring chemical tolerance of the solvents to these substrates and products. A second challenge will be sufficient and economical recovery of solvent from particles to ensure the costs of solvent loss are negligible. Another challenge will be scaling and matching the rates and scales of capture and mineralisation streams to ensure one process does not dwarf the other. It should be noted that these processes will entail significant logistical hurdles to clear in order to deliver millions of tons substrates (likely by rail), and where to ship and store the carbonaceous products.

The answer to this question may come from the economic drivers for mineralisation. The present costs of transport and sequestration of CO_2_ are approximately $20 (USD) per tonne CO_2_, in addition to a cost of capture target of $30 (USD) per tonne CO_2_, which at present is economically unfeasible to make a profit without incentives or a market for mineralisation. Sequestration in the US could provide a revised $85 (USD) per tonne CO_2_ 45Q, mineralisation could be profitable at a reasonable $25 (USD) per tonne. As stated above, these margins are yet enough to entice broad commercialization, but could be made to be if other markets and incentives can be made available *via* an integrated capture and mineralisation approach. Bourgeois makes a compelling case that precipitated carbonates and silicates can be recovered and sold as binding agents, mineral aggregates, and concrete for commercial construction applications providing a further economic incentive for capture and permanent sequestration.^101^ If materials like this could be sold as concrete constitutes, there could be a sizable reduction in the carbon footprint of the construction industry, which Liu estimates to be more than 4 billion tonnes on CO_2_.^96^

## Needs and outlook

Our vision is to use solvent-based processes to convert point-source emission sites to factories that manufacture a myriad of CO_2_-containing products adaptable to market needs. We are at the turning point where we can continue to use 20th century monolithic capture and conversion infrastructure or we can begin the transition to a new 21st century paradigm of integrated solvent-based carbon capture and conversion technologies (Fig. 1).

To make this vision a reality, there are many areas of need. First, more studies of known reactivity of CO_2_ in viable carbon capture solvents is needed to identify viable solvents, catalysts, reagents and products that could impact the three time periods identified above, including studies of solvent durability and lifetime for utilization reactions. Second, we have to expand the product base, which can only occur from the design and realization of new reactivities of captured CO_2_. Also, one can envision further integration approaches where the processes are in a single unit, where utilization can be used to influence VLE in the absorber, and thus further drive the absorption. Third, processes should assess modular microchannel reactors that can run in parallel for varied products. Fourth, recent advances in additive manufacturing opens new doors for advanced heat integration enabling more efficient integrated systems. Lastly, comprehensive TEA and life cycle analysis (LCA) studies will be needed to qualify and quantify the full range of benefits of integration in order to produce policy-relevant evidence to co-create the legislative landscape to enable the at-scale deployment of these technologies.

## Conclusions

In this contribution, we have made a case for how solvent-based carbon capture can be integrated with conversion as a key element of the transition to a net-zero emission economy. Carbon dioxide utilization has always been a lofty target for at-scale production of green CO_2_-derived fuels, chemicals and materials, as these materials would command enough revenue to pay for capture, with further incentives from tax credits. The key to making CO_2_-derived materials and products market competitive is by integration of solvent-based CO_2_ capture and conversion. We have envisioned how solvents could be adapted for the differing needs for capture, conversion, and mineralisation in the near, intermediate, and long term. We have identified white space and research needs in this emerging field to achieve success. Ultimately, integration of capture and conversion represents promising approach to achieve global net-zero emissions.

## Author contributions

Dr Heldebrant provided conceptualization, supervision, writing. Dr Kothandaraman, Professor Mac Dowell and Ms Brickett provided investigation and writing.

## Conflicts of interest

There are no conflicts to declare.

## Supplementary Material
